# Enhanced Photoelectrocatalytic Performance of ZnO Nanowires for Green Hydrogen Production and Organic Pollutant Degradation

**DOI:** 10.3390/ma18020444

**Published:** 2025-01-19

**Authors:** Nawal Al Abass, Talal F. Qahtan, Amani M. Alansi, Almqdad Bubshait, Maria Al-Ghamdi, Zahra Albu, Noof Soltan Albasiry, Hisham Mohammed Aljahfal, Abdulrahman E. Aldossary, Mohammed Tariq Faraj

**Affiliations:** 1 King Abdulaziz City for Science and Technology (KACST), Hydrogen Technologies Institute, Mailbox 6086, Riyadh 11442, Saudi Arabia; abubshait@kacst.gov.sa (A.B.); haljahfal@kacst.gov.sa (H.M.A.); aealdossary@kacst.gov.sa (A.E.A.); 2 Physics Department, College of Science and Humanities in Al-Kharj, Prince Sattam Bin Abdulaziz University, Al-Kharj 11942, Saudi Arabia; 3 Chemistry Department, College of Science, Taiz University, Taiz 12372, Yemen; mn.alansi2016@gmail.com; 4 College of Engineering Sciences, Materials Science and Engineering Department, King Fahd University of Petroleum and Minerals, Dhahran 31261, Saudi Arabia; marialghamdi3@gmail.com; 5 The Center of Excellence for Advanced Materials and Manufacturing, King Abdulaziz City for Science and Technology, Riyadh 11442, Saudi Arabia; zalbu@kacst.gov.sa; 6 Institute for Materials Discovery, Functional Materials and Energy Device Group, University College London, London WC1E 6BT, UK; 7 Department of Physics, College of Science, Princess Nourah Bint Abdulrahman University, Riyadh 84428, Saudi Arabia; noof.ss.b@gmail.com; 8 King Abdulaziz City for Science and Technology (KACST), Microelectronics and Semiconductors Institute, Mailbox 6086, Riyadh 11442, Saudi Arabia; mfaraj@kacst.gov.sa

**Keywords:** ZnO nanostructures, anodic oxidation, photoelectrocatalysis, water oxidation, waste water treatment, surface defects

## Abstract

With growing environmental concerns and the need for sustainable energy, multifunctional materials that can simultaneously address water treatment and clean energy production are in high demand. In this study, we developed a cost-effective method to synthesize zinc oxide (ZnO) nanowires via the anodic oxidation of zinc foil. By carefully controlling the anodization time, we optimized the Zn/ZnO-5 min electrode to achieve impressive dual-function performance in terms of effective photoelectrocatalysis for water splitting and waste water treatment. The electrode exhibited a high photocurrent density of 1.18 mA/cm^2^ at 1.23 V vs. RHE and achieved a solar-to-hydrogen conversion efficiency of 0.55%. A key factor behind this performance is the presence of surface defects, such as oxygen vacancies (OVs), which enhanced charge separation and boosted electron transport. In tests for waste water treatment, the Zn/ZnO-5 min electrode demonstrated the highly efficient degradation of methylene blue (MB) dye, with a reaction rate constant of 0.4211 min^−1^ when exposed to light and a 1.0 V applied voltage significantly faster than using light or voltage alone. Electrochemical analyses, including impedance spectroscopy and voltammetry, further confirmed the superior charge transfer properties of the electrode under illumination, making it an excellent candidate for both energy conversion and pollutant removal. This study highlights the potential of using simple anodic oxidation to produce scalable and efficient ZnO-based photocatalysts. The dual-function capability of this material could pave the way for large-scale applications in renewable hydrogen production and advanced waste water treatment, offering a promising solution to some of today’s most pressing environmental and energy challenges.

## 1. Introduction

The global push for sustainability and environmental conservation has created an urgent demand for technologies that can address multiple challenges simultaneously, particularly in energy generation and environmental remediation, two areas intrinsically linked to climate change, resource depletion, and pollution. The pursuit of sustainable energy sources, such as green hydrogen, combined with the need for efficient waste water treatment, has driven research into multifunctional materials capable of tackling both issues in tandem [1,2,3,4]. A particularly promising approach lies in the development of materials that can perform dual functions: catalyzing water oxidation (water splitting) to produce oxygen and hydrogen while simultaneously degrading pollutants in waste water. This dual functionality not only combines energy production with environmental cleanup in a single, integrated system but also enhances overall efficiency and reduces costs [5,6]. The necessity for such multifunctional materials arises from the limitations of traditional methods of energy production and waste management, which are often resource-intensive, environmentally harmful, and unsustainable in the long term.

In this context, zinc oxide (ZnO) has emerged as a highly promising semiconductor for photoelectrocatalysis due to its excellent photoactivity, chemical stability, and non-toxic nature. These properties make ZnO an ideal candidate for applications in both environmental protection and energy generation. The wide bandgap of ZnO (approximately 3.37 eV) allows it to effectively absorb UV light, generating electron–hole pairs that drive catalytic reactions like water oxidation and pollutant degradation. Moreover, its high electron mobility and strong exciton binding energy enhance charge carrier separation, which is crucial for improving the efficiency of photocatalytic processes [7,8,9,10]. Traditional methods for synthesizing ZnO nanostructures, including chemical vapor deposition, sol–gel processes, solvothermal and hydrothermal synthesis, and heterojunction fabrication, are well-established techniques known for yielding well-defined and highly controlled ZnO morphologies [11,12,13,14,15,16,17,18]. However, these methods often involve complex procedures, require high temperatures, and depend on expensive precursors, which can limit their scalability and practical application.

The anodic oxidation of zinc foil offers a simplified, cost-effective approach for fabricating ZnO nanostructures. This method involves the electrochemical oxidation of zinc foil in a controlled environment, directly forming ZnO on the surface of foil. The key advantages of anodic oxidation include lower processing temperatures, the use of inexpensive materials, and the ability to form ZnO nanostructures with a high surface area and well-ordered crystalline. Unlike traditional synthesis methods, anodic oxidation is straightforward and allows for precise control over the layers of ZnO layer thickness and morphology by adjusting the anodization time and conditions [19,20,21,22]. While anodic oxidation is an established technique, its application in developing ZnO nanostructures for dual-function photoelectrocatalytic systems—particularly for water oxidation and waste water treatment—represents a novel and impactful use of this method.

In this study, we introduce a novel, cost-effective method for creating high-performance ZnO nanostructures by precisely controlling the anodic oxidation of zinc foil. Through the careful adjustment of anodization time, we successfully developed ZnO nanowires with enhanced surface area, improved crystallinity, and an optimal defect density, particularly oxygen vacancies (VOs), which play a crucial role in enhancing the photoelectrochemical performance of material. These engineered defects significantly boost charge separation and improve photocatalytic efficiency. The optimized Zn/ZnO-5 min electrode demonstrated excellent photocurrent density and solar-to-hydrogen conversion efficiency, making it highly effective for both water oxidation and waste water treatment, particularly in degrading organic dyes like methylene blue (MB). What distinguishes this work is its integration of energy production and environmental remediation into a single, scalable system. This dual-functional ZnO photoelectrode not only advances our understanding of ZnO nanostructures but also addresses critical challenges in renewable energy and environmental protection. By demonstrating the scalability and practical application of anodic oxidation for producing high-performance ZnO photoelectrocatalysts, this study sets a new standard in the field and opens up pathways for large-scale applications in green hydrogen production and efficient waste water treatment.

## 2. Experimental Section

### 2.1. Materials and Chemicals

Zinc foil (Zn) (99.9% purity, Sigma-Aldrich, St. Louis, MI, USA) was utilized as the starting material for the anode. Platinum mesh (Pt) (Goodfellow, Delson, QC, Canada) served as the cathode. The electropolishing solution consisted of 85 wt% phosphoric acid (Sigma-Aldrich, St. Louis, MI, USA) mixed with ethanol (Merck, Ames, IA, USA) in a 1:2 volume ratio. Sodium hydroxide (NaOH, Sigma-Aldrich, St. Louis, MI, USA) and ammonium chloride (NH_4_Cl, Sigma-Aldrich, St. Louis, MI, USA) were employed as an electrolyte solution in the anodizing process. Deionized water was used throughout the procedure.

### 2.2. Cleaning and Preparation of Zn Foil

Zinc foils were cut into the desired dimensions and placed in a 1:1 mixture of ethanol and acetone. The foils were subjected to ultrasonication for 15 min to remove surface contaminants. Following this, the foils were thoroughly rinsed with deionized water and allowed to air dry for 10 min.

### 2.3. Electropolishing of Zn Foil

The electropolishing process was performed in a two-electrode system, where the Zn foil served as the anode and the platinum mesh as the cathode. The electrodes were immersed in an electropolishing solution composed of 85 wt% phosphoric acid and ethanol (1:2 vol.). The distance between the electrodes was maintained at 3 cm, and the solution temperature was controlled at 5 ± 0 °C using the temperature control system. A constant voltage of 10 V was applied across the electrodes using the DC power supply, with vigorous stirring maintained throughout the 10 min polishing process. After electropolishing, the Zn foil was rinsed with deionized water and dried using a gentle stream of high-purity nitrogen gas to prevent oxidation or contamination.

### 2.4. Anodic Oxidation to Fabricate Zn/ZnO Nanowire Electrodes

**Scheme 1:**
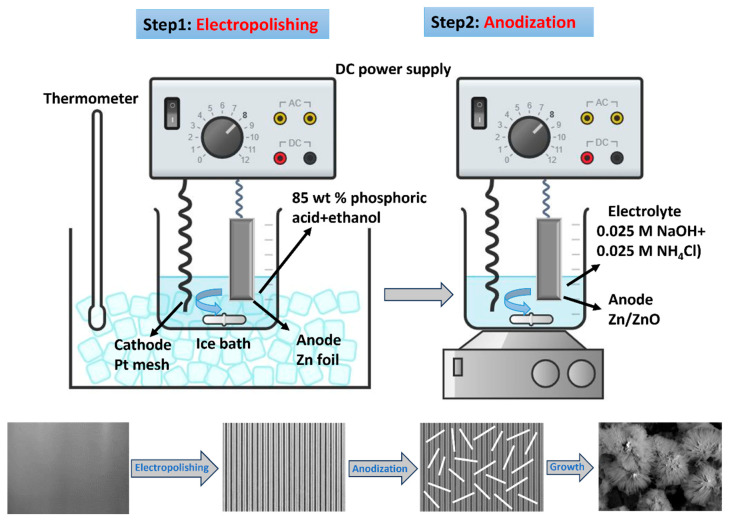
A visual representation of the experimental setup used for electrochemical anodization to create ZnO nanowires from ZnO foil.

Following electropolishing, the Zn foil underwent anodic oxidation in an electrolyte solution composed of 0.025 M NaOH and 0.025 M NH_4_Cl dissolved in deionized water. The electropolished Zn foil was positioned as the anode, and a platinum mesh was used as the cathode, maintaining a 3 cm distance between them. The electrolyte temperature was kept constant at 5 ± 0 °C using the temperature control system. A constant voltage of 10 V was applied using the DC power supply to induce anodization. The anodization process was conducted for varying durations of 1 min, 5 min, and 10 min to study the effect of time on the growth of ZnO nanowires. After each anodization step, the Zn foil was thoroughly rinsed with deionized water and dried using nitrogen gas. The electrodes were categorized and labeled based on the specific treatments they underwent to ensure clarity in the subsequent analyses. The untreated zinc foil was labeled as “Zn foil”, the electropolished electrode as “EP Zn foil”, and the anodized electrodes as “Zn/ZnO-1 min”, “Zn/ZnO-5 min”, and “Zn/ZnO-10 min”, corresponding to anodization durations of 1, 5, and 10 min, respectively. These labels were used consistently throughout this study to facilitate a comparison of the different conditions. Scheme 1 illustrates the process of electrochemical anodization used to produce ZnO nanowires from ZnO foil in a laboratory environment.

### 2.5. Photocatalytic Measurements for Degradation of Organic Waste Water

The photoelectrochemical (PEC) degradation of organic waste water was investigated using the Zn/ZnO-5 min electrode in a PEC cell. The cell featured a three-electrode setup with the Zn/ZnO electrode as the anode, a Ag/AgCl reference electrode, and a platinum mesh cathode. The electrodes were placed in a photocell with a quartz window to allow for the precise control of light exposure, with the anode and cathode spaced 5 cm apart to ensure consistent electric field distribution. The photocatalytic properties were evaluated using a Xenon lamp with an intensity of 100 mW/cm^2^ (provided by Newport Corporation, Irvine, CA, USA), chosen for its ability to simulate sunlight. The Zn/ZnO-5 min electrode was immersed in a 10 ppm methylene blue (MB) solution, a standard model contaminant, and stirred in the dark for 30 min to reach adsorption–desorption equilibrium. This pre-treatment step was critical for ensuring consistent interaction between the dye molecules and the electrode surface. After equilibrium was reached, the lamp was activated, and three different treatment methods were tested: (1) exposure to light only, (2) application of 1 V vs. RHE, and (3) combined light exposure with 1 V vs. RHE. The degradation of MB was monitored using UV-Vis spectrometry by measuring the absorption peak at regular intervals, allowing for a detailed comparison of the photo-degradation efficiency under different treatment conditions. To evaluate the photocatalytic performance of Zn/ZnO-5 min electrode in dye degradation, the ratio of the MB dye concentration at a specific time *t* to the initial concentration (*C*/*C*_0_) is commonly used. This ratio provides a normalized measure of the degradation progress where *C*_0_ is the initial concentration of the dye before irradiation (at *t* = 0). *C* is the concentration of the dye at a specific time t during or after irradiation. This ratio is particularly useful for kinetic analyses of the degradation process under a pseudo-first-order kinetic model, which is often applicable to photocatalytic degradation. It can be described by the following formula:

ln(*C*_0_/C) = *kt*

where *k* is the apparent rate constant for the degradation reaction. *t* is the irradiation time.

### 2.6. Photoelectrochemical Measurements for Water Oxidation

To evaluate the photoelectrochemical water splitting performance of the Zn/ZnO electrodes, Linear Sweep Voltammetry (LSV), chronoamperometry, and Electrochemical Impedance Spectroscopy (EIS) were conducted using a PGSTAT204 Autolab system (Metrohm) controlled by Nova 2.1 software. All experiments were performed in a quartz photocell filled with a 0.5 M Na_2_SO_4_ electrolyte solution, commonly used in water splitting studies, employing a three-electrode configuration. The Ag/AgCl electrode served as the reference electrode, ensuring consistent potential measurements, while a large-area platinum mesh electrode was used as the counter electrode, providing excellent conductivity and stability. The photocell was illuminated with light at an intensity of 100 mW/cm^2^ to simulate solar conditions. The LSV measurements were recorded at a scan rate of 50 mV/s, providing detailed insights into the onset potential and current density of the Zn/ZnO electrodes. These measurements are crucial for understanding the efficiency of the water splitting process and the photocatalytic activity of the material. For the EIS analysis, a potential of 0.5 V vs. the Reversible Hydrogen Electrode (RHE) was applied. A small sinusoidal perturbation with a 10-millivolt amplitude was superimposed, and the response was measured across a frequency range from 0.1 Hz to 100 kHz. This technique allowed for the assessment of charge transfer resistance and other key electrochemical parameters. EIS spectra were obtained both in the dark and under illumination to evaluate the photoelectrochemical performance under realistic operating conditions. All potentials were referenced to the RHE, with the Ag/AgCl reference electrode calibrated appropriately to ensure accuracy. The RHE potential (E(RHE)) was calculated using the following formula: E(RHE) = E(Ag/AgCl) + 0.241 + 0.059 pH.

### 2.7. Structural Characterization

The ZnO nanowires formed on the surface of the Zn foil were characterized using a comprehensive set of techniques to evaluate their morphological, structural, and chemical properties. Scanning Electron Microscopy (SEM) was performed using a Tescan Lyra-3 (Brno, Czech Republic) instrument to assess the surface morphology of the electrodes, focusing on the size, shape, and distribution of the nanowires. X-ray diffraction (XRD) analysis was conducted using a Rigaku (Park, TX, USA) International X-ray diffractometer to determine the crystal structure and phase purity of the ZnO nanorods, with measurements taken over a 2θ range of 20–80°. In addition to these techniques, X-ray Photoelectron Spectroscopy (XPS) was carried out using a Thermo Scientific (Waltham, MA, USA) K-alpha XPS system. This technique provided detailed information about the chemical composition and electronic states of the elements present in the ZnO nanowires, offering insights into surface chemistry and the oxidation states of zinc and oxygen.

## 3. Results and Discussion

### 3.1. Structure of Zn/ZnO Electrodes

**Figure 1:**
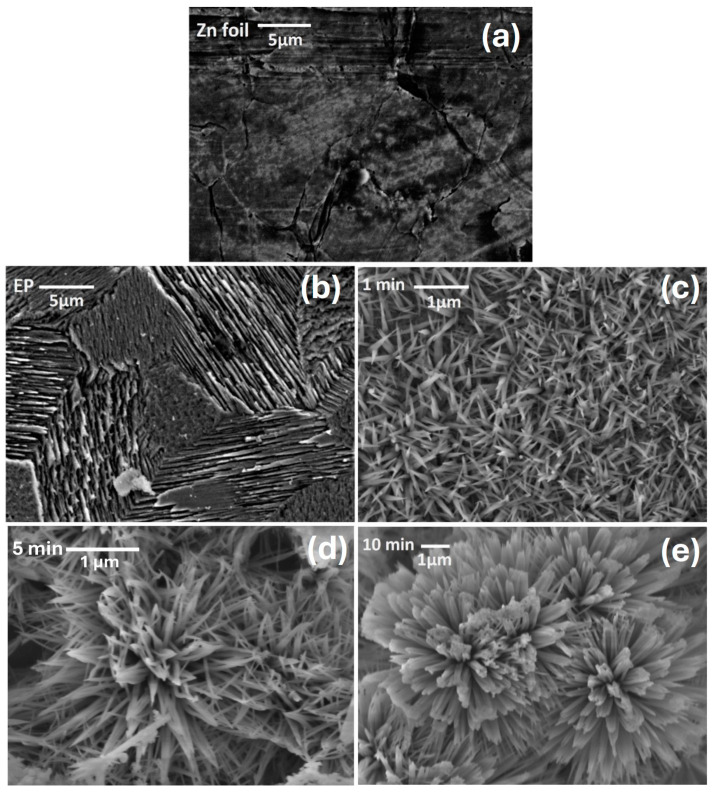
SEM images showing the surface morphology evolution of Zn foil during anodization. (**a**) Zn foil. (**b**) Electropolished Zn foil. (**c**) Zn/ZnO-1 min. (**d**) Zn/ZnO-5 min. (**e**) Zn/ZnO-10 min.

The surface morphology of the fabricated Zn/ZnO electrodes, analyzed using SEM (Figure 1), reveals the structural evolution of ZnO nanostructures on a Zn foil substrate during different stages of preparation and anodization. Initially, the untreated Zn foil (Figure 1a) exhibits a relatively smooth surface with minor cracks and defects, characteristic of metallic zinc, and no discernible nanostructures or ZnO layer. Following electropolishing (Figure 1b), the surface becomes cleaner and more refined, displaying linear grooves and striations that result from the polishing process, which removes surface oxides and irregularities to prepare the foil for uniform nanostructure growth during anodization. As anodization progresses, significant morphological changes occur. After 1 min (Figure 1c), small, uniformly distributed ZnO nanowires or needle-like formations begin to appear, marking the initial stage of ZnO nucleation and offering increased surface area coverage. By 5 min (Figure 1d), the ZnO nanostructures have grown into well-defined, elongated nanowires with uniform density, forming a robust and highly structured layer. This stage represents an optimal balance between structural integrity and high surface area, making it ideal for photocatalytic applications. After 10 min (Figure 1e), the nanostructures evolve into hierarchical, flower-like morphologies composed of interconnected nanowires or nanorods. These complex structures provide increased porosity and surface complexity, making them suitable for advanced applications, such as enhanced photocatalysis or sensing [16,23].

The SEM analysis effectively illustrates the morphological progression from the untreated Zn foil to the hierarchical structures formed after extended anodization, demonstrating the ability to control ZnO nanostructure growth for tailored applications. The 5 min stage is optimized for high surface area uses, while the 10 min stage offers hierarchical designs beneficial for multifunctional applications.

**Figure 2:**
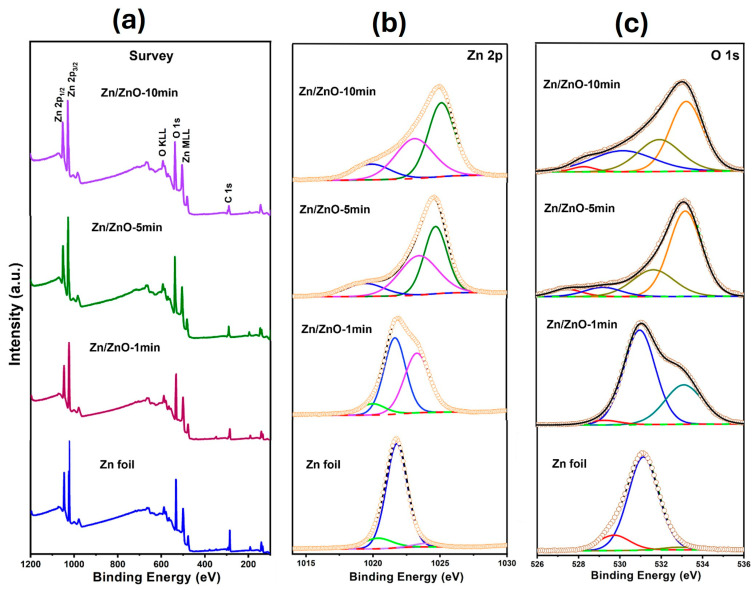
XPS of Zn foil and Zn/ZnO electrodes as mentioned in the figures. (**a**) Survey, (**b**) high-reclusion Zn 2p, (**c**) and high-reclusion O 1s.

The XPS spectra presented in Figure 2 provide detailed insights into the chemical states and surface compositions of Zn foil and Zn/ZnO electrodes after anodic oxidation for 1, 5, and 10 min. The survey spectra at Figure 2a offer a comprehensive snapshot of the elemental composition of each sample. In the survey spectra (Figure 2a), the Zn 2p, O 1s, and C 1s peaks are clearly visible across all samples, confirming the presence of zinc and oxygen elements. For the Zn foil, sharp peaks for Zn 2p, Zn MLL (Auger peak), and C 1s stand out, typical of bare metallic zinc. After anodization (Zn/ZnO-1 min, 5 min, 10 min), the intensity of the Zn 2p and O 1s peaks grows, signaling the formation of ZnO. Additionally, the oxygen-related peaks (O 1s and O KLL) become more prominent, confirming the thickening of the oxide layer with time. The C 1s peak, likely from surface contamination, is still visible across all spectra. As anodization proceeds, the spectra shift from being dominated by metallic zinc to showing increasingly strong ZnO signatures, confirming successful oxidation over time.

In the high-resolution Zn 2p spectra (Figure 2b), we can clearly observe the chemical transformation of zinc. The Zn foil spectrum displays sharp and symmetrical Zn 2p peaks, characteristic of metallic Zn, with no signs of oxidation. However, after anodization (Zn/ZnO-1 min, 5 min, 10 min), the Zn 2p peaks broaden and shift, reflecting the transition from metallic Zn (Zn^0^) to oxidized Zn (Zn^2+^) in ZnO. Peak fitting reveals the presence of Zn^2+^ and possible defects like interstitial zinc, which increase in intensity as anodization progresses. Over time, the Zn 2p peaks evolve from pure metallic Zn to a mix of Zn^0^ and Zn^2+^, indicating the formation of ZnO and associated defect states [23,24].

The O 1s high-resolution spectra (Figure 2c) provide insights into the oxygen species present on the surface. For Zn foil, the narrow peak primarily reflects surface-adsorbed oxygen species, such as hydroxyl groups, due to the minimal oxide layer. As anodization advances (Zn/ZnO-1 min, 5 min, 10 min), the O 1s peak broadens and becomes more complex. Peak fitting reveals lattice oxygen (O^2−^) around 529–530 eV, confirming ZnO formation. At higher binding energies (531–533 eV), peaks correspond to OVs, surface hydroxyl groups, and adsorbed water [16,24,25]. These peaks intensify with longer anodization times, indicating the growth of surface defects and a thicker ZnO layer. The O 1s spectra confirm the increasing presence of lattice oxygen and defect-related species, such as hydroxyl groups and OVs, as anodization continues [23,24].

The shift in the lattice oxygen peak to lower binding energy in the Zn/ZnO-5 min electrode, compared to Zn foil, indicates changes in the chemical environment due to the formation of a more uniform, defect-rich ZnO layer. The rise in high-density peaks at higher binding energies with extended oxidation times suggests an increase in surface hydroxyl groups and adsorbed water, likely from the larger surface area and higher defect density in the ZnO nanostructures. These defects significantly enhance photoelectrochemical performance by promoting charge separation and improving electron transport [26].

The XPS data provide a clear progression of surface chemistry, with Zn foil transitioning to a defect-rich ZnO layer. As anodization continues, the ZnO layer becomes increasingly pronounced with more defects (OVs and hydroxyl groups), which are key to boosting the material’s photoelectrochemical capabilities for applications like water splitting or photocatalysis.

**Figure 3:**
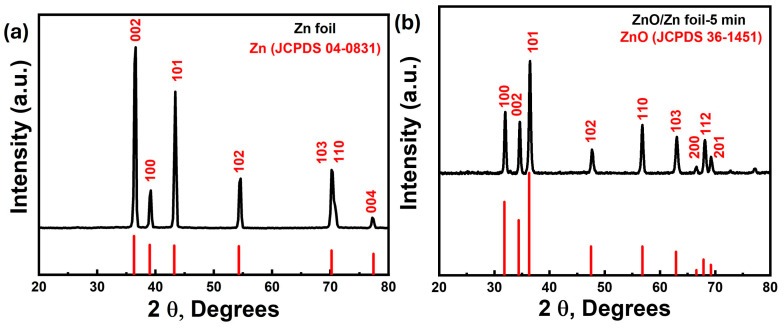
XRD of (**a**) Zn foil and (**b**) Zn/ZnO-5 min.

To assess the crystal structure and confirm the successful formation of ZnO on Zn foil, we performed XRD analysis on the Zn/ZnO-5 min electrode and compared the results with those of the untreated Zn foil. Figure 3 presents a comparison of the XRD patterns before and after 5 min of anodic oxidation. The analysis clearly demonstrates the formation of a ZnO layer on the Zn foil, evidenced by the emergence of new diffraction peaks corresponding to ZnO phases. In Figure 3a, the XRD pattern of the pristine Zn foil shows sharp peaks associated with specific crystal planes (101), (100), (002), (102), (103), and (110), reflecting the high purity and crystalline nature typical of the hexagonal close-packed (HCP) structure of metallic zinc [27,28]. After anodic oxidation, Figure 3b reveals additional peaks in the Zn/ZnO-5 min electrode, which correspond to the hexagonal wurtzite structure of ZnO [29]. These new peaks confirm the formation of a ZnO layer on the surface of Zn foil, while the persistence of the original Zn peaks indicates that the underlying foil remains structurally intact. Additionally, the inclusion of the XRD standard patterns for Zn (JCPDS 04-0831) [30] and ZnO (JCPDS 36-1451) [31] in the figures provides a clear reference for phase identification. The variations in peak intensities and positions between the two patterns underscore the structural changes due to the ZnO phase development.

### 3.2. PFC Performance of Zn/ZnO Electrodes

The PEC kinetics at the ZnO anode/electrolyte interface were evaluated using EIS at 0.5 V vs. RHE under 100 mW/cm^2^ light intensity. The corresponding Nyquist plots for the prepared anodes revealed significant insights into the PEC performance. The EIS data demonstrated a pronounced sensitivity to light, as evidenced by changes in the arc radius of the Nyquist plots, which reflect variations in junction resistance between the ZnO anodes and the electrolyte. An equivalent circuit model, incorporating solution resistance (Rs) and charge transfer resistance (Rct), was used to analyze these plots. A smaller arc radius in the Nyquist plot under illumination indicates enhanced charge carrier mobility, leading to reduced Rct and improved electron–hole separation. Additionally, lower R values suggest better electrolyte conductivity, which further enhances electron transfer and overall water splitting efficiency [16,26,32].

**Figure 4:**
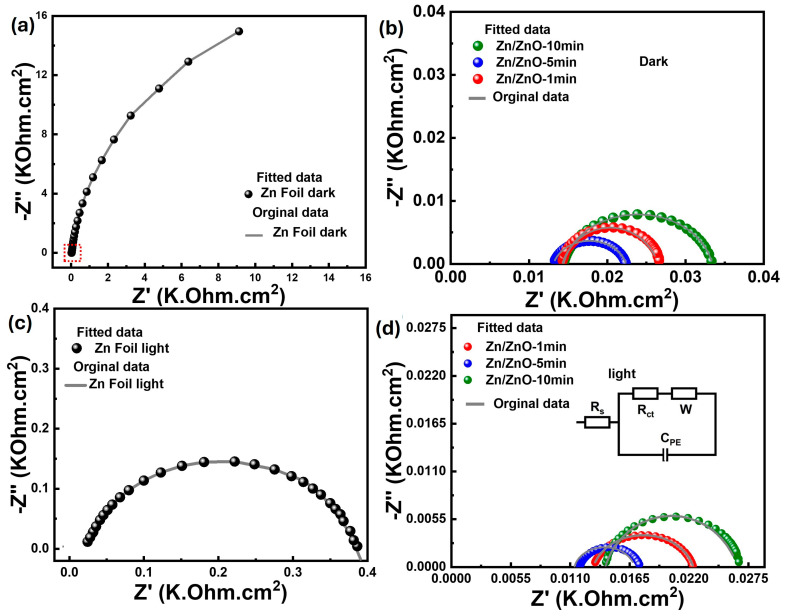
Nyquist plots from EIS of Zn foil and Zn/ZnO electrodes under dark and illuminated conditions (100 mW/cm^2^). (**a**) Zn foil in the dark, (**b**) The selected red area in figure a is magnified as Zn/ZnO electrodes anodized for 1, 5, and 10 min in the dark, (**c**) Zn foil under illumination, (**d**) Zn/ZnO electrodes under illumination, and the insert provides a suitable illustration of the EIS, as outlined by the Randles circuit model.

Figure 4 presents four Nyquist plots that assess the Rct and other electrochemical properties of Zn foil and Zn/ZnO electrodes under both dark and illuminated conditions. In Figure 4a, the Nyquist plot for Zn foil in the dark reveals a large semicircle, indicative of high Rct and poor electrochemical activity due to limited charge transfer in the absence of light. Conversely, Figure 4b shows Nyquist curves for Zn/ZnO electrodes anodized for 1, 5, and 10 min in the dark. The Zn/ZnO-5 min electrode displays the smallest semicircle, reflecting the lowest Rct and superior electrochemical performance, while the Zn/ZnO-10 min electrode exhibits the largest semicircle, suggesting that prolonged anodization negatively impacts performance.

Figure 4c shows that under illumination, the Nyquist plot for Zn foil reveals a reduced semicircle compared to dark conditions, indicating lower Rct and improved electrochemical activity, although it remains less effective than the Zn/ZnO electrodes. Figure 4d illustrates the EIS response of Zn/ZnO electrodes under illumination at 100 mW/cm^2^, with semicircles generally smaller than those observed in dark conditions, signifying enhanced charge transfer and lower Rct under light exposure. The Zn/ZnO-5 min electrode consistently exhibits the smallest semicircle, confirming its superior electrochemical performance, while the Zn/ZnO-10 min electrode continues to show the largest semicircle, highlighting the detrimental effects of excessive anodization.

The exceptional performance of the Zn/ZnO-5 min sample, marked by the lowest Rct and Rs, is likely due to an optimal balance of surface properties, including passivation layer thickness and surface roughness, which collectively enhance charge transfer efficiency [32,33]. Furthermore, XPS analysis indicated that surface defects play a crucial role in facilitating charge separation and improving electron transport in these samples. However, as observed in the Zn/ZnO-10 min sample, excessive anodization leads to increased Rct due to the formation of thicker passivation layers that hinder charge transfer by extending the electron travel distance [34]. These findings underscore the critical importance of optimizing anodization time to achieve superior photoelectrochemical properties, with surface defects contributing positively to the overall performance of the Zn/ZnO-5 min electrode. The estimated values of Rct and Rs for the Zn/ZnO electrodes are summarized in Table 1, providing a clear comparison of their performance characteristics.

**Figure 5:**
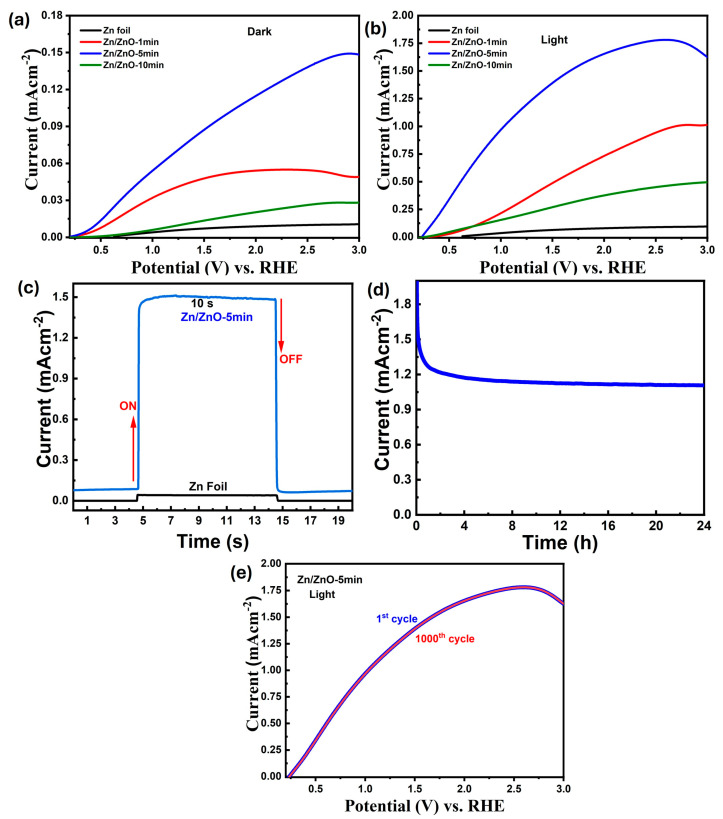
Photoelectrochemical performance of Zn/ZnO electrodes prepared under different anodization times. (**a**) LSV curves in dark conditions, (**b**) LSV curves under illumination (100 mW cm^−2^), (**c**) chronoamperometric response at 1.6 V vs. RHE under light on/off cycles, (**d**) long-term stability test under continuous illumination at 100 mW cm^−2^, and (**e**) cyclic stability of the Zn/ZnO-5 min electrode.

To evaluate the photoelectrochemical performance of the Zn/ZnO electrodes for water photoelectrolysis, LSV was employed. Upon absorption of simulated sunlight, the ZnO material in the photoanode is excited, generating electrons in the conduction band and holes in the valence band. The application of an external voltage drives these electrons towards the Pt foil counter electrode, where they reduce protons to produce hydrogen, while the holes at the anode facilitate water oxidation, releasing oxygen. Figure 5 illustrates the photoelectrochemical behavior of the Zn/ZnO electrodes under various conditions. In Figure 5a, LSV curves for Zn foil and Zn/ZnO electrodes analyzed for 1, 5, and 10 min in the dark show low current densities across all samples due to the absence of light. Despite this, the Zn/ZnO-5 min sample exhibits the highest current density, suggesting a superior performance even without illumination. Under illuminated conditions, as depicted in Figure 5b, all samples show significantly higher current densities, with the Zn/ZnO-5 min electrode standing out, reaching approximately 1.18 mA/cm^2^ at 1.23 V vs. RHE. This robust photocatalytic activity is likely attributed to the unique morphology of the Zn/ZnO-5 min nanowires, which provide a larger surface area for light absorption and more active sites for photoconversion. This underscores the critical role of optimizing semiconductor morphology to enhance photoelectrochemical properties.

The chronoamperometric response of the Zn/ZnO-5 min electrode, shown in Figure 5c, further supports these findings. When illuminated at a constant potential of 1.6 V vs. RHE, the current density rapidly increases to around 1.48 mA/cm^2^, demonstrating a strong and stable photocurrent. The current quickly drops to near-zero when the light is turned off, confirming the photocurrent’s dependence on light exposure. This rapid response to light, even at a lower potential than the LSV tests, highlights the electrode’s efficiency and its practical applicability in photoelectrochemical processes, such as water splitting. The long-term stability of the Zn/ZnO-5 min electrode is demonstrated in Figure 5d. Under continuous illumination at 100 mW/cm^2^, the current density shows a slight initial decrease but stabilizes at approximately 1.15 mA/cm^2^, maintaining consistent photoelectrochemical activity over a 24 h period. Additionally, a cyclic stability test (Figure 5e) was performed, revealing no observable changes in performance before and after multiple cycles, further highlighting the electrode’s excellent durability. These results confirm the Zn/ZnO-5 min film’s ability to sustain a high photocurrent and exhibit remarkable photostability during water photo-oxidation over extended periods, making it a reliable and durable candidate for long-term applications.

The LSV and EIS results are interlinked, offering a comprehensive understanding of the Zn/ZnO-5 min electrode’s superior performance. While LSV highlights its impressive photocurrent generation and light responsiveness, EIS measurements provide insights into the underlying electrochemical processes, revealing low Rct and optimal charge carrier mobility under illumination. Notably, the XPS analysis suggests that surface defects, particularly OVs, play a crucial role in this enhanced performance by facilitating charge separation and improving electron transport. Together, these results underscore the effectiveness of the Zn/ZnO-5 min electrode in maximizing photoelectrochemical efficiency through a finely tuned balance of structural and electronic properties [23,26]. The PEC performance of the optimized Zn/ZnO film is compared with previously reported ZnO electrodes in the literature, as shown in Table 2. This comparison focuses on photoanodes exposed to AM 1.5 G sunlight and similar illumination conditions. Notably, the Zn/ZnO-5 min film demonstrates a photocurrent density of 1.18 mA/cm^2^ at 1.23 V vis RHE, surpassing the performance of most ZnO photoanodes reported in earlier studies. This significant achievement underscores the potential of the Zn/ZnO-5 min electrode for advanced photocatalytic applications.

**Figure 6:**
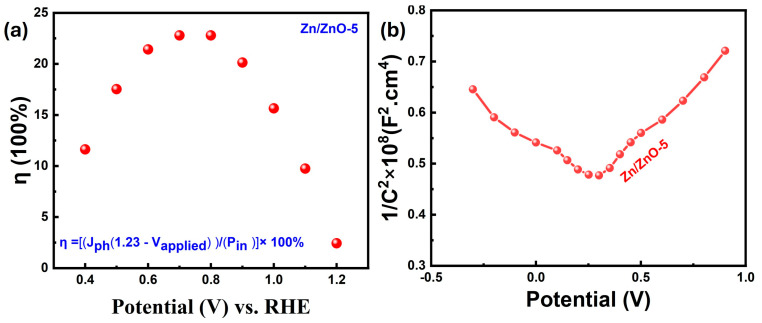
(**a**) Photoconversion efficiency (η) versus applied potential (V) for the Zn/ZnO-5 min sample. (**b**) Mott–Schottky plot of the Zn/ZnO-5 min electrode.

The solar-to-hydrogen (STH) conversion efficiency, a crucial metric for evaluating the effectiveness of solar energy conversion into hydrogen fuel, was calculated by determining the photoconversion efficiency (η) of the Zn/ZnO photoanodes. The efficiency was computed using the following formula [39]:

$$\eta =\left[\frac{Jph(1.23―Vapplied)}{Pin}\right]\times 100\%$$

where *Jph* represents the photocurrent density (in mA/cm^2^), *Vapplied* is the applied external potential (in V), and *Pin* denotes the incident light power (in mW/cm^2^), providing a clear quantitative measure of the photoanode’s performance. The standard reversible redox potential for water electrolysis is 1.23 V, which serves as a critical reference point for these calculations. In the broader context of ZnO-based photoanodes, STH conversion efficiencies typically range from 0.1% to 1.0% [15,39,40,41]. The highest recorded efficiency for Au@Pt-modified ZnO/CdS reaches 0.655% at an applied potential of ~1.0 V [41]. This range reflects the inherent challenges in optimizing material properties such as bandgap, charge carrier mobility, and surface defect engineering to improve photocatalytic performance. Notably, our study demonstrated that the Zn/ZnO-5 min sample achieved an impressive STH efficiency of 0.55% at a lower applied potential of 0.7 V, setting a new benchmark in the field without the need for complex metal mixtures. The efficiency curve, as shown in Figure 6a, peaks at approximately 0.55% at 0.7 V vs. RHE, establishing this as the optimal potential for maximizing hydrogen production efficiency. Beyond this point, the efficiency diminishes, underscoring the precise operating range where the photocatalyst performs most effectively.

The Mott–Schottky analysis in Figure 6b reveals the semiconductor type and provides key insights into carrier concentration and flat-band potential, both crucial for optimizing photoelectrochemical performance. The V-shaped plot for the Zn/ZnO-5 min electrode indicates the formation of a p-n junction between the Zn foil and the newly formed ZnO layer, which significantly enhances the separation of photogenerated electron–hole pairs under illumination, leading to a more efficient charge transfer, vital for water splitting [42]. This p-n junction also suggests increased carrier concentration, likely due to surface defects like oxygen vacancies introduced during anodization. These defects are critical for improving photoelectrochemical efficiency by acting as electron traps, reducing recombination, and facilitating charge transport. The enhanced performance observed in the EIS, LSV, and STH efficiency analyses further highlights the importance of surface defect engineering, particularly oxygen vacancies, in optimizing ZnO-based photoanodes for advanced photoelectrochemical applications.

### 3.3. Photocatalytic Performance for Degradation of Organic Waste Water

**Figure 7:**
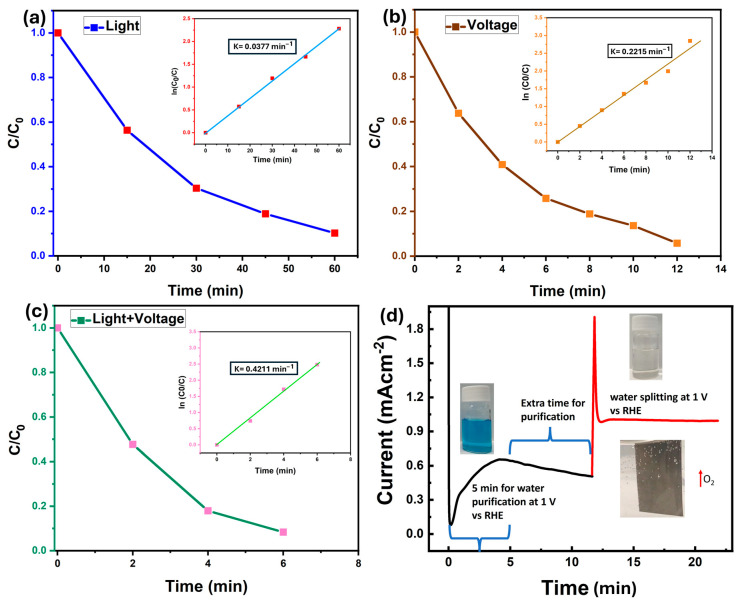
MB degradation and kinetic analysis (**a**) with light only, (**b**) with voltage only, and (**c**) MB degradation under combined light and 1.0 V vs. RHE over 5 min. (**d**) The plot shows the current density (mA/cm^2^) over time during a photoelectrochemical process, where the Zn/ZnO electrode is used for simultaneous water purification and water splitting.

Given that the Zn/ZnO-5 min electrode demonstrated superior performance in the various measurements conducted in this study, it was selected over the other electrodes for the assessment of photocatalytic and photochemical performance. The photocatalytic performance of Zn/ZnO-5 min electrodes was assessed by degrading MB [43,44], a toxic and carcinogenic dye commonly found in industrial waste water, under three different conditions: light exposure, applied voltage, and their combination (Figure 7). The observed color change from blue to nearly colorless confirmed the successful breakdown of the dye. This figure highlights the enhanced contaminant degradation and the efficiency of water splitting using a photocatalytic or photoelectrocatalytic system. The comparison across light-only, voltage-only, and combined light and voltage conditions demonstrates that combining these effects significantly accelerates the degradation process. Furthermore, Figure 7d showcases the system’s dual functionality in both water purification and water splitting, underscoring its potential for environmental and energy applications. Figure 7a depicts photocatalysis under light and the degradation of the MB (C/C_0_) over time under light exposure. The steady decrease in contaminant concentration over 60 min indicates effective degradation. The inset plot shows a linear relationship in a plot of ln(C_0_/C) versus time, with a rate constant *k* = 0.0377 min^−1^, suggesting first-order reaction kinetics under light irradiation [44]. Figure 7b shows the electrocatalysis under voltage and the contaminant degradation over time under an applied voltage. The faster reduction in concentration compared to Figure 7a suggests that the applied voltage significantly enhances the degradation process. The inset shows a higher first-order rate constant *k* = 0.2215 min^−1^, indicating more efficient degradation under voltage alone.

Figure 7c illustrates the combined effect of light and voltage and the degradation of the contaminant when both light and voltage are applied simultaneously. The contaminant concentration decreases much more rapidly than in Figure 7a or Figure 7b, revealing a synergistic effect when both light and voltage are used together. The inset shows a significantly higher rate constant *k* = 0.4211 min^−1^, highlighting the enhanced efficiency of the degradation process under combined conditions. Figure 7d presents water splitting and purification and current density (mA/cm^2^) as a function of time during a photoelectrochemical water splitting experiment, presumably conducted at 1.0 V vs. RHE. Initially, the system is used for water purification for the first 6 min, as evidenced by a gradual increase in current density. After this period, the process continues to further purify the water. Around the 12 min mark, the system transitions to water splitting mode, resulting in a significant spike in current density, which corresponds to the generation of oxygen bubbles (O_2_) on the electrode surface. This spike indicates active water splitting, producing hydrogen on the counter electrode. Overall, Figure 7 effectively illustrates the enhanced performance of the Zn/ZnO-5 min electrodes under different operational conditions and underscores the versatility and potential of the system in both environmental remediation and energy production. The photocatalytic performance of the optimized Zn/ZnO-5 min film is compared with previously reported ZnO-based photocatalysts, as summarized in Table 3. This comparison emphasizes the degradation of MB dye as a model pollutant, the type of light source employed, and the degradation rate constant. Notably, the Zn/ZnO-5 min film demonstrates a significantly higher degradation rate, surpassing the performance of ZnO-based photocatalysts reported in earlier studies. These results underscore the exceptional potential of the Zn/ZnO-5 min electrode for advanced photocatalytic applications, particularly in the field of water treatment.

### 3.4. Proposed Mechanism for Photoelectrochemical and Photocatalytic Performance

The degradation of MB dye by Zn/ZnO-5 min is highly efficient due to the combined effects of photocatalysis and electrochemical reactions, particularly when surface defects like oxygen vacancies are present, as these enhance charge separation and improve electron transport. The degradation process under different conditions of light only, voltage only, and combined light and 1.0 V vs. RHE can be explained through distinct mechanisms, with simultaneous oxygen evolution occurring at the anode.

#### 3.4.1. Light Only

When only light is applied, MB degradation is driven primarily by photocatalysis, but the process is relatively slow. The absorption of light by ZnO excites electrons, leading to the generation of electron–hole pairs (Equation (1)). However, in the absence of an applied voltage, the separation of these charge carriers is less efficient, resulting in a higher rate of recombination (Equation (2)). This recombination reduces the number of free charge carriers available for redox reactions, thereby slowing down the degradation process. Nonetheless, some of the holes that reach the ZnO surface can still participate in the oxidation of MB or in the generation of reactive oxygen species (ROS), such as hydroxyl radicals (Equation (3)). These ROS, along with the holes, contribute to the breakdown of MB (Equation (4)). However, the lower efficiency in generating ROS and holes, due to the lack of applied voltage, results in a gradual reduction in the MB concentration, reflecting a slower degradation over 60 min. Additionally, without the applied voltage, there is no significant oxygen evolution, and the process depends entirely on the photocatalytic activity of ZnO for MB degradation [50,51].

ZnO + *h_V_* → *e_CB_*^−^ + *h_V__B_*^+^ (1)

*e_CB_*^−^ + *h_V__B_*^+^ → Recombination (2)

*h_V__B_*^+^ + H_2_O → ^•^OH + H^+^ (3)

MB + *h_V__B_*^+^/^•^OH → Degraded Products (4)

#### 3.4.2. Voltage Only

In the absence of light, MB degradation under an applied voltage occurs primarily through electrochemical reactions at the electrode surfaces rather than through photocatalytic processes. The applied voltage facilitates oxidation reactions at the ZnO anode, where MB molecules are directly oxidized without the contribution of light-induced electron–hole pairs (Equation (5)). This oxidation process breaks down MB molecules, though with lower efficiency compared to light-assisted photocatalysis, due to the lack of additional charge carriers generated by light. Concurrently, reduction reactions at the cathode create a more reductive environment, which can indirectly support the degradation process (Equation (6)). Without light to generate further charge carriers, the degradation of MB relies entirely on these electrochemical reactions driven by the applied voltage. While the absence of photocatalytic effects leads to a slower overall degradation compared to combined light and voltage conditions, it is more efficient than degradation with light alone due to the direct influence of electrochemical processes. This results in a moderate decrease in MB absorbance within 10 min [52].

MB → Oxidized MB + *e*^−^ (5)

MB + *e*^−^ → Reduced MB (6)

#### 3.4.3. Combined Light and 1.0 V vs. RHE

**Figure 8:**
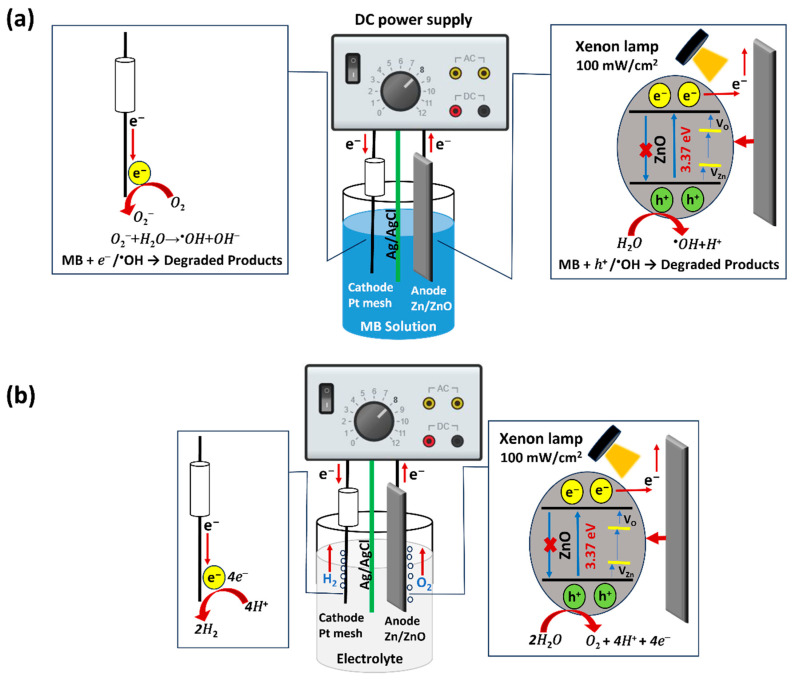
Mechanism of (**a**) MB dye degradation and (**b**) subsequent water splitting Zn/ZnO-5 min electrode in a PEC setup under simulated sunlight (100 mW/cm^2^) and applied voltage 1 V vs. RHE.

When the Zn/ZnO-5 min electrode is exposed to simulated sunlight in an electrochemical cell with an applied voltage of 1 volt, the following photoelectrochemical processes efficiently degrade the MB dye (Figure 8a). The ZnO absorbs photons (*h_V_*), exciting electrons (*e*^−^) from the valence band to the conduction band, leaving behind holes (*h*^+^) (Equation (7)). The 1-volt applied voltage enhances the separation of these electron–hole pairs by driving the electrons toward the cathode and retaining the holes at the anode, thus minimizing recombination. At the cathode (Pt), the electrons reduce dissolved oxygen to produce superoxide anion, which further reacts to produce hydroxyl radicals (Equations (8) and (9)). At the anode (Zn/ZnO-5 min), the holes directly oxidize the MB dye or water molecules to produce hydroxyl radicals (Equation (10)).

The highly reactive hydroxyl radicals (^•^OH) then degrade the MB dye into harmless byproducts such as CO_2_, H_2_O, and mineral acids. The applied voltage of 1 volt accelerates the degradation process by facilitating the separation of charge carriers and promoting the formation of ROS, which are crucial for the rapid breakdown of MB dye [53,54,55].

ZnO + *h_V_* → ZnO (*e*^−^ + *h*^+^) (7)

O_2_ + *e*^−^ → O_2_^−^ (8)

O_2_^−^ + H_2_O → ^•^OH + OH^−^ (9)

H_2_O + h^+^ → ^•^OH + H^+^ (10)

After MB degradation, the resultant solution, now free from organic contaminants, is used for water oxidation with the same Zn/ZnO-5 min electrode under simulated sunlight and a 1.0 V applied voltage (Figure 8b). As before, ZnO absorbs photons, generating electron–hole pairs, and then the applied voltage ensures effective separation, with electrons driven to the cathode and holes remaining at the anode. The holes at the anode oxidize the water molecules (Equation (11)). This reaction produces oxygen gas (O_2_) at the anode. Simultaneously, the electrons reduce protons at the cathode, producing hydrogen gas (Equation (12)) [23,26,29,53].

2H_2_O + 4*h*^+^ → O_2_ + 4H^+^ (11)

2H + 2*e*^−^ → H_2_ (12)

The application of a 1.0 V potential is crucial for enhancing the photoelectrochemical performance of the Zn/ZnO-5 min electrode. This voltage accelerates water oxidation by providing the necessary energy for holes to efficiently oxidize water while ensuring a continuous flow of electrons to the cathode, vital for efficient hydrogen production. Additionally, it optimizes charge carrier separation, promotes ROS generation, and drives the redox reactions essential for pollutant breakdown and hydrogen and oxygen production. The defects in the Zn/ZnO-5 min structure further improve charge separation and electron transport, significantly boosting overall efficiency.

The Zn/ZnO-5 min electrode, synthesized via a simple and cost-effective anodic oxidation method without noble metals or complex treatments, offers excellent accessibility and scalability. Its dual functionality in hydrogen production and pollutant degradation underscores its potential for renewable energy and environmental remediation. Table 4 provides a comprehensive comparison with other dual-function photocatalysts, highlighting its competitive efficiency, simplicity, and versatility.

## 4. Conclusions

In this study, we successfully demonstrated a novel and cost-effective approach to fabricating ZnO nanostructures through the controlled anodic oxidation of Zn foil, leading to significant advancements in photoelectrochemical applications. The Zn/ZnO-5 min electrode, optimized through the precise control of anodization time, exhibited remarkable photocatalytic and photoelectrochemical performance, making it a highly promising candidate for both water oxidation and waste water treatment. The surface defects, particularly OVs and zinc interstitials, played a pivotal role in enhancing charge separation and electron transport, which were corroborated by extensive EIS, LSV, and XPS analyses. This study not only underscores the critical importance of optimizing structural and electronic properties in ZnO nanostructures but also sets a new benchmark for developing multifunctional materials that can contribute to sustainable energy conversion and environmental protection. This research paves the way for scalable and practical applications in green hydrogen production and efficient pollutant degradation, showcasing the dual functionality of the Zn/ZnO-5 min electrode as a powerful tool in addressing some of the most pressing challenges in renewable energy and environmental remediation.

## Figures and Tables

**Table 1:** The values of Rct and Rs for the Zn/ZnO electrodes.

Electrode	Rs (K·Ohm·cm^2^) in Dark	Rct (K·Ohm·cm^2^) in Dark	Rs (K·Ohm·cm^2^) in Light	Rct (K·Ohm·cm^2^) in Light
Zn foil	0.1	10	0.02	0.36
Zn/ZnO-1 min	0.0144	0.0186	0.013	0.0087
Zn/ZnO-5 min	0.0133	0.0087	0.001	0.0055
Zn/ZnO-10 min	0.0143	0.0123	0.0143	0.0123

**Table 2:** Comparison of PEC performances of ZnO electrodes reported in the literature, including nanostructured thin films fabricated using physical, chemical, and chemo-physical methods.

Photoanodes	Preparation Method	I (mA cm^−2^) @ 1.23 V vs. RHE	Electrolyte	Light Source	Ref.
ZnO nanorods	Chemical synthesis	0.705	0.5 M Na_2_SO_4_	AM 1.5 G (100 mW/cm^2^)	[35]
ZnO nanowires	Chemical bath deposition	0.15	0.1 M NaOH	UV lamp (11.5 mW/cm^2^)	[36]
ZnO nanorods	Dip coating	0.39	0.5 M Na_2_SO_4_	Xe lamp (100 mW/cm^2^)	[37]
ZnO thin films	Sol–gel spin coating	0.005 at 1.00 V	0.5 M Na_2_SO_4_	500 W Xe lamp + AM 1.5 G filter (UV-Vis-NIR) 100 mW/cm^2^	[14]
ZnO thin films	DC magnetron sputtering	0.05	0.5 M Na_2_SO_4_	AM 1.5 G solar	[38]
ZnO nanowires	Hydrothermal	0.03	0.1 M Na_2_S + 0.2 M NaOH	500 W Xe lamp	[16]
ZnO Nanorod array films	Hydrothermal	0.28	0.1 M Na_2_SO_4_	AM 1.5 G (100 mW/cm^2^)	[15]
ZnO nanowires with oxygen vacancies	Electrochemical method	1.2	0.5 M Na_2_SO_4_	AM1.5 G, 100 mW/cm^2^	[23]
ZnO nanorods films with oxygen vacancies	Oxidation of Zn films	1.14	0.5 M Na_2_SO_4_	AM1.5 G, 100 mW/cm^2^	[26]
ZnO nanowires with oxygen vacancies	Anodic oxidation	1.18	0.5 M Na_2_SO_4_	AM1.5 G, 100 mW/cm^2^	This work

**Table 3:** Comparison of MB dye degradation by ZnO-based photocatalysts reported in the literature.

Photocatalysts	Preparation Method	Degradation Rate Constant Min^−1^	Light Source	Ref.
ZnO/Co_3_O_4_	Liquid deposition	1.74	A xenon lamp (300 W, λ = 320–1100 nm)	[45]
ZnO nanostructures	Decomposition of ZnC_2_O_4_·2H_2_O	0.043	18 W Black light	[46]
ZnO nanostructures	Ultrasound-assisted hydrolysis	0.0209	10 W UV light	[47]
ZnO superstructures	Thermal decomposition of zinc nitrate	80% degradation in 180 min	120 W UV light	[48]
ZnO nanoparticles	Sol–gel	0.0084	UV lamp (Philips, 12 W)	[49]
Zn/ZnO-5 min	Anodic oxidation	0.4211	AM1.5 G, 100 mW/cm^2^	This work

**Table 4:** A comprehensive assessment comparison with the most common dual photocatalysts.

Catalyst	Hydrogen Production (mA/cm^2^ at 1.23 V vs. RHE)	Dye Degradation Rate Constant min^−1^	Stability	Ref.
TiO_2_	1.87 (doped with Au) at E/0.187 V and bare TiO_2_ (0.28 at the same E)	0.006	Not mentioned	[56]
g-C_3_N_4_	0.1258	0.04411	~20,000 s and no change in photocurrent	[57]
BiVO_4_	0.14	0.00545	1 h and no change in photocurrent	[58]
CdS	~1.49 mA/cm^2^ at 1 V vs. Ag/AgCl	0.01	Not mentioned	[59]
Zn/ZnO	1.18	0.4211	24 h and no change in photocurrent	This work

## Data Availability

The data supporting the findings of this study are available within the article.
